# The yearly pattern of *Ashworthius sidemi* Schulz, 1933 infection in cervids: a 10-year study in southern Poland and northern Slovakia

**DOI:** 10.2478/jvetres-2026-0037

**Published:** 2026-06-30

**Authors:** Jerzy Kowal, Anna Wyrobisz-Papiewska, Weronika Rynkiewicz, Marek Wajdzik, Paweł Nosal

**Affiliations:** 1 Department of Zoology and Animal Welfare, University of Agriculture in Kraków, 30-059 Kraków, Poland; 2 Department of Forest Biodiversity, University of Agriculture in Kraków, 31-425 Kraków, Poland

**Keywords:** *Ashworthius sidemi*, diagnostic sensitivity, faecal egg shedding, seasonal dynamics, stage structure

## Abstract

**Introduction:**

The widespread distribution of the blood-sucking parasitic species *Ashworthius sidemi* among cervids highlights the need for increased understanding of how to prevent its transmission to livestock. Such knowledge should include detailed data on its life cycle, which may provide insights into the pattern of invasion and guide control measures.

**Material and Methods:**

Adult and subadult specimens of *A. sidemi* were obtained by necropsy examinations of 108 naturally infected cervids between 2011 and 2021. The month of host collection was used to divide the obtained data into two periods of the year: warm (May to October) and cold (November to March). The differences in the overall mean intensity of infection within a particular month and between periods were statistically analysed. Molecular analyses were conducted to confirm species identification.

**Results:**

A total of 16,574 specimens were found. The mean intensity of infection for adults and for subadults differed significantly, both within a particular month and between the two periods of the year (P-value < 0.01). Correspondence analysis revealed two distinct groups based on the month of collection from the host and the increased occurrence of adults and/or subadults in the abomasum. The occurrence of adult specimens was the greatest during the warm periods of the year, with their first appearance recorded in May.

**Conclusion:**

Although infection intensity remains relatively stable throughout the year, eggs shedding may be influenced by the occurrence of adults, what depends on the period of the year.

## Introduction

*Ashworthius sidemi* Schulz, 1933 was originally described in sika deer (*Pseudaxis hortulorum*, syn. *Cervus nippon*) from the mainland of north-eastern Asia (22). However, it has since spread to various European regions and been identified in most wild ruminant host species (4, 5). This invasive species is assumed to be capable of infecting domestic ruminants grazing in areas shared with wild hosts (5). Experimental studies have shown that sheep are particularly susceptible to *A. sidemi* infection (13). Therefore, understanding how to prevent transmission of this parasite to livestock is crucial (4, 11). *Ashworthius sidemi*, like *Haemonchus contortus*, belongs to the Strongylida order, the Trichostrongyloidea superfamily and the Haemonchinae subfamily (1). The life cycle of *A. sidemi* should be thoroughly studied, including the conditions affecting its development, in a manner similar to how the life cycle of closely related species such as *Haemonchus contortus* has been studied, because it also parasitises domestic ruminants (2). The specific objective of the present study was to demonstrate whether the pattern of *A. sidemi* invasion can be characterised based on *post mortem* examinations of infected cervids.

## Material and Methods

### Necropsy examination and parasite identification

Adult and subadult specimens of *Ashworthius sidemi* were obtained from the abomasum contents and washings of the abomasal mucosa of naturally infected hosts (n = 108). Necropsy examinations were conducted over the period from 2011 to 2021 on 65 roe deer, 37 red deer and 6 fallow deer. These hosts were harvested during hunting seasons or collected as roadkill following parasitological procedures (10), with a modification involving the use of 1 mm mesh sieve for rinsing of abomasal contents. The origin of the hosts was restricted to southern Poland and northern Slovakia, regions characterised by a temperate, warm and transitional climate where the range of mean annual precipitation is approximately 550–1400 mm in Małopolskie voivodeship and 600–1700 mm in adjacent areas of Slovakia. All hosts were categorised based on the month of parasite collection and subsequently divided with reference to that month into periods according to the average daily air temperature. The months from April to October were classified as the warm period (with an average temperature of 8.8–17.9°C and initial temperature of 5.5°C at the turn of March and April), while the months from November to March were classified as the cold period (with an average temperature of −1.5–4°C and initial temperature of 6.9°C at the turn of October and November).

The main distinguishing features for identifying *A. sidemi* were the shape, length and size of the oesophageal tooth (4, 22). To differentiate adults from subadults, all males and females were categorised based on body length (9). Specimens were mounted on glass slides in glycerol jelly or lactophenol (7) and subjected to detailed morphological analyses. The following characteristics were inspected: the development of the bursa copulatrix and the proportion of ventral to lateral lobes, the stage of sclerotisation of spicules for males; and the development of the ovijector, the proportion of ovijector length to body width at the vulva region, the number of eggs in the oviducts and presence of vulva sealing after copulation for females. During the necropsies, specimens of other members of the Haemonchinae subfamily, *Haemonchus contortus*, were also collected and identified (14).

### Molecular analysis

Genomic DNA was extracted from a morphologically analysed *Ashworthius sidemi* nematode (a single female specimen from a red deer) using a method based on the Sherlock AX kit (A&A Biotechnology, Gdańsk, Poland), with additional mechanical lysis of the sample performed using a scalpel.

The second internal transcribed spacer (ITS-2) of the ribosomal DNA (rDNA) was used as the target to define a specific marker. This spacer and partial flanking 5.8S and 28S regions were amplified using the 5’-TGTGTCGATGAAGAGCGCAG-3’ forward and 5’-TGGTTAGTTTCTTTTCCTCCGC-3’ reverse primers. The PCR mix was comprised of 25 μL of PCR Mix Plus (A&A Biotechnology), 0,2 μM of each primer and 5 μL of genomic DNA. The PCR conditions were as follows: a single step of pre-denaturation at 94°C for 120 s; 10 cycles of 94°C for 15 s, 47°C for 30 s and 72°C for 1 min; 30 cycles of 94°C for 15 s, 56°C for 30 s and 72°C for 1 min; and a final extension at 72°C for 5 min. The purified PCR products were sequenced (Macrogen, Amsterdam, the Netherlands), analysed with the CLC Main Workbench 22 package (Qiagen, Hilden, Germany), and compared with sequences available in GenBank using the BLAST program (rRNA/ITS database). The new nucleic acid sequence was deposited in GenBank.

### Statistical analysis

The differences in the overall mean intensity of infection (*i.e*. both adults and subadults) within a particular month and between periods of the year were analysed using the non-parametric Kruskal–Wallis ANOVA in the Statistica package v. 13 (Dell, Round Rock, TX, USA). The same analysis was performed to compare the mean intensity of infection exclusively for adults and exclusively for subadults.

Correspondence analysis was conducted using Centurion XVIII software (Statgraphics, The Plains, VA, USA) to analyse the relationship between the period of the year and the increased occurrence of adults or subadults of *Ashworthius sidemi* in the abomasum of infected hosts. The mean intensity of infection for adults and subadults within a particular month was used as the measure characterising the association between the rows and columns in the contingency table.

## Results

### Necropsy examination and parasite identification

The results of the necropsies are summarised in Table 1. A total of 16,574 specimens of *Ashworthius sidemi* were found, comprising 6,594 adults and 9,980 subadults. Notably, more than two-thirds of the total specimens (11,489) were collected during the cold season, with subadults predominating (9,016). The majority of specimens were recorded in hosts sampled in December and January, when the monthly subtotals were 5,203 and 4,302, respectively. In addition to *A. sidemi*, 866 specimens of *Haemonchus contortus* were identified. Co-infection with both parasite species was observed in 16 hosts, including 1 red deer and 15 roe deer. The intensity of *A. sidemi* invasion varied among host species and ranged between 2 and 1,600 in roe deer, 2 and 390 in fallow deer and 1 and 1765 in red deer, with mean intensities of 181, 93 and 118, respectively.

**Table 1.** Specimens of *Ashworthius sidemi* derived from cervids during post mortem examinations

Period	Necropsy month	Hosts with infection (n)	Hosts with co-infection (n)	Mean intensity of infection	Adult-parasite mean intensity	Subadult-parasite mean intensity
Hot	May	7	3	106	87	32
Hot	June	10	3	95	94	1
Hot	July	6	1	200	200	2
Hot	August	9	3	176	107	105
Hot	September	6	-	42	35	12
Hot	October	7	1	51	56	7
Cold	November	11	-	174	60	161
Cold	December	24	3	217	175	143
Cold	January	26	1	165	19	162
Cold	March	2	1	40	0	40

For female specimens of *A. sidemi*, body length was not a reliable criterion for distinguishing adults from subadults, as some very small individuals were already fully mature and classified as adults. This phenomenon was particularly prominent in specimens collected from roe deer during the warm period of the year (May to August). Exceptionally small and thin specimens were also observed in red deer harvested in December. Conversely, subadults collected from roe deer in January were notably long and thick.

### Molecular analysis

Molecular analysis confirmed the morphologically based species identification. The newly obtained sequence (GenBank accession No. PX735851) showed 99.44–100% similarity to the reference species (*A. sidemi* OM443078.1 and EF467325.1).

### Statistical analysis

No significant differences in the overall mean intensity of infection within a particular month or between periods of the year, were noted. However, the mean intensities of infection only for adults and only for subadults differred significantly both month-to-month and inter-period at P-value < 0.01.

**Fig. 1.**
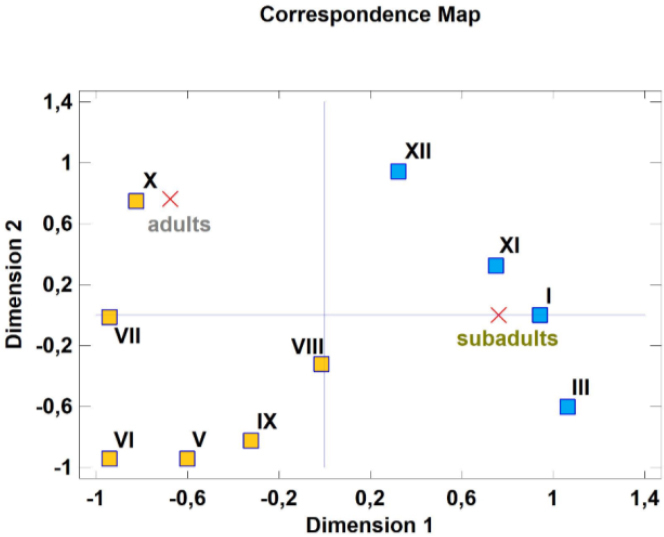
Connection between the period of the year and the increased occurrence of adults and/or subadults of *Ashworthius sidemi* in the abomasa of infected cervid hosts (symmetric map of the correspondence analysis). Red crosses – type of specimens; yellow squares – months from the warm period of the year (V – May, VI – June, VII – July, VIII – August, IX – September and X – October); blue squares – months from the cold period of the year (I – January, III – March, XI – November and XII – December

**Fig. 2.**
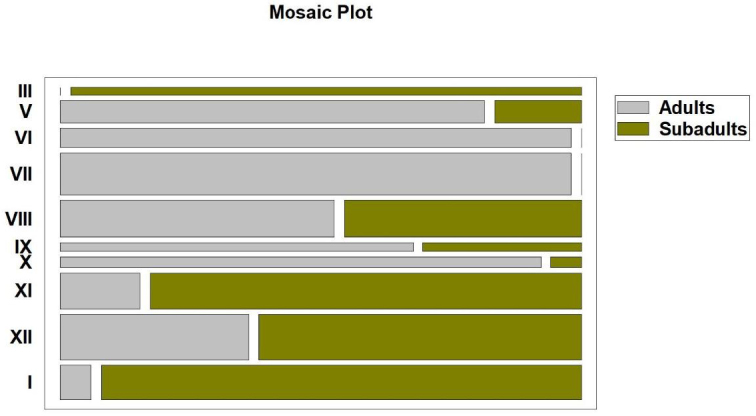
Relationships between the particular month and proportions of adults and of subadults of *Ashworthius sidemi* in the abomasa of infected cervid hosts. Length of the rectangles corresponds to the strength of the correlation; width corresponds to the number of observations. III – March; V – May; VI – June; VII – July; VIII – August; IX – September; X – October; XI – November; XII – December; I – January

Both the correspondence map (Fig. 1) and the mosaic plot (Fig. 2) explain almost 100% of the variability amongst the rows and columns. The correspondence analysis divided the month of host collection and the increased occurrence of adults and/or subadults in the abomasum into two clearly distinct groups (Fig. 1). Based on the results, it can be stated that the occurrence of *Ashworthius sidemi* adult specimens was greatest during the warm period of the year. The distance between October and adults on the correspondence map is the shortest (Fig. 1), indicating that this month was most strongly associated with their presence in the host abomasum. Other months (*i.e*. May, June, July, August and September) showed a weaker association. In the case of subadults, their occurrence in the cervid abomasum was clearly linked to all months in the cold period of the year, among which January showed the strongest association, as the distance between cross and square is the shortest (Fig. 1). However, the distance observed for November appears comparable.

The increased occurrence of *Ashworthius sidemi* adult specimens during the warm period of the year was also evident in the mosaic plot (Fig. 2). The plot shows that adults first appeared in May and their proportion became greater in June and July. This dominance was most pronounced in July, as indicated by the largest area corresponding to adults. In August, the proportion of adults was nearly equal to that of subadults, after which it gradually increased again from September to October. At the beginning of the cold period of the year (*i.e*. in November), subadults began to dominate to varying degrees, ultimately peaking in March.

## Discussion

The morphological identification of *Ashworthius sidemi* Schulz, 1933 was formerly complicated by the occurrence of *Ashworthius gagarini* Kostyaev, 1969 in nematode systematics. Initially, two species of the *Ashworthius* genus were recorded in cervids in Europe: *A. sidemi* and *A. gagarini* (9). Morphological differences between the species were minor and included dimensions, arrangement and proportions of certain structures, *e.g*. uterine branches, vulva and bursal rays. However, Dróżdż *et al*. (5) and Ferté *et al*. (8) concurred that the description of *A. gagarini* was in fact a description of subadult *A. sidemi*. Thus, in European cervids there is only one species of the genus causing infections, *A. sidemi*, and its detailed descriptions allow adults to be accurately distinguished from subadults.

The intensity of infection remained relatively constant throughout the year, as no statistically significant differences were observed; however, seasonal variation occurred in the proportions of subadults and adults, which varied with the period of the year. The presence of adults was associated with the warm period of the year (from April to October), whereas subadults were more commonly observed during the cold period (from November to March). Considering the near-total dominance of adults observed in June and July, it can be assumed that cervid acquisition of invasive larvae at the end of winter and during spring (from February to April) either did not occur or occurred only on a limited scale. This is likely because of the prepatent period, which lasts approximately two months in the warm period of the year and up to four months in the cold period (15). The subadults observed in May were likely remnants of the autumn infection. Those appearing in July were third-stage larvae collected in May or June. The number of eggs cannot be directly inferred from the number of females, but rather depends on external factors, both endogenous and exogenous. Although July, August and October corresponded to larger proportions of adults, linking these periods to greater egg production and to egg shedding increase is speculative. During the remainder of the year, egg shedding is likely to be drastically reduced; however, because larval stages and egg shedding were not directly observed, this interpretation is also speculative. It may be hypothesised that a self-cure phenomenon could potentially contribute (in November), but further research including coproscopic investigation would be needed to substantiate this.

The results above are consistent with those obtained from marals observed in the natural habitat of *Ashworthius sidemi* (15, 20). Adult specimens were found in abomasum at the end of the winter; however, they were not observed around December and January, suggesting that a self-cure phenomenon likely occurred in the autumn.

Our predicted peak of egg shedding coincides with that observed in marals in coproscopic studies conducted in the Altai region (6). The same authors reported a sharp decline in the number of eggs shed in November and December, which may suggest a similar timing of self-cure. Slightly different results were obtained by Magdálek *et al*. (16) in molecular studies of larvae recovered from fallow deer faeces. They indicate that under the climatic conditions of Central Europe, egg shedding remains stable from April to January, reflecting a shift in the seasonal pattern under the new conditions of *A. sidemi* occurrence. In contrast, our findings demonstrate that such a shift does not occur in the studied cervid hosts.

*Ashworthius sidemi* has never been detected in necropsy studies of domestic ruminants in Europe, which may suggest an absence of transmission from cervid hosts. To date, its presence has only been reported in molecular analyses of faecal nematode larvae samples (18). However, we concur with Rehbein *et al*. (21) that definitive evidence of transmission should be based on the recovery of the parasite during necropsy.

Our results, together with those of Lyubimov (15) and Ovcharenko (20), may explain why the *A. sidemi* invasion has not penetrated domestic ruminant populations. The period of increased parasite egg production in cervids does not coincide with their presence on pastures used by domestic ruminants, limiting the possibility of indirect transmission *via* shared grazing areas. Considering that red deer are the principal host of *A. sidemi*, the primary risk is associated with roe deer populations that occur in areas of permanent sympatry with red deer (23). Under the traditional grazing schedules of the temperate-climate zone (mid-April to mid-October, or mid-May to mid-September in mountain regions), this risk remains low. However, ongoing climate change – with its associated extension of the vegetation and, consequently, grazing periods – may substantially increase the potential for transmission. Prolonged grazing not only elevates the probability of *A. sidemi* infection but is also epizootiologically unfavourable, as it intensifies the spring emergence of *Haemonchus* sp. infections.

When comparing our results with those extrapolated by Hoberg (11) from domestic hosts for *Haemonchus contortus*, it can be inferred that the observed intensities of *A. sidemi* infection are unlikely to substantially affect morbidity or mortality in native cervids. The typical hosts of *A. sidemi* are Asiatic cervids, primarily the sika deer (*Cervus nippon* Temminck, 1838) (5). Consequently, among European species, infection levels in red deer are expected to be comparatively lower because of the close phylogenetic relationship between the parasite’s original and novel host species (21). In roe deer, however, the parasite–host association is evolutionarily more recent, which likely contributes to the higher infection intensities observed.

European bison (*Bison bonasus*) is a protected and conservation-relevant species in which *A. sidemi* infection has been documented in Poland. The first case was reported in a free-living herd in the Bieszczady Mountains in south-eastern Poland (5), and was followed by detection in a captive herd in the Białowieża Forest in the north-east of the country (4). Over the years, a evident increase in intensity of infection was observed, which was later followed by a gradual decline (3, 12). This trend is probably associated with the progressive adaptation of the host population to the novel parasite. Among other free-ranging ungulates, the Tatra chamois (*Rupicapra rupicapra tatrica*) may be a species for which *A. sidemi* has high pathogenic potential, given that infection would represent a completely novel parasite–host relationship (11). Although *A. sidemi* infection has not yet been detected in the Tatra chamois, it has been recorded in Alpine chamois introduced to lower-montane areas (17, 19). This pattern may reflect ecological constraints limiting spillover, potentially reinforced by the seasonality of *A. sidemi* egg shedding and the only partial overlap between the vertical habitat ranges of red deer and the Tatra chamois.

## Conclusion

The results of the present study demonstrate that the pattern of *Ashworthius sidemi* invasion can be reliably characterised through necropsy examinations of infected cervids. Infection intensity remained stable throughout the year, with seasonal variation occurring only in the proportion of adults versus that of subadults. These findings suggest that egg shedding followed a pattern similar to that observed in cervids from endemic regions. Limited temporal overlap between periods of peak shedding in cervids and the presence of domestic ruminants on shared pastures may partially explain the absence of *A. sidemi* transmission to those ruminants. The seasonality of *A. sidemi* egg production in cervid hosts has practical implications for the translocation of domestic ruminants, as it reduces the sensitivity of coproscopic detection. The predominance of subadult stages during the colder months would likely further decrease diagnostic efficiency, making coproscopic examinations of translocated or imported animals during winter largely ineffective because of near-zero or substantially reduced egg-per-gram values.
